# Pectolinarigenin Induced Cell Cycle Arrest, Autophagy, and Apoptosis in Gastric Cancer Cell via PI3K/AKT/mTOR Signaling Pathway

**DOI:** 10.3390/nu10081043

**Published:** 2018-08-08

**Authors:** Ho Jeong Lee, Venu Venkatarame Gowda Saralamma, Seong Min Kim, Sang Eun Ha, Suchismita Raha, Won Sup Lee, Eun Hee Kim, Sang Joon Lee, Jeong Doo Heo, Gon Sup Kim

**Affiliations:** 1 Research Institute of Life science and College of Veterinary Medicine, Gyeongsang National University, 501 Jinju-daero, Jinju 52828, Korea; lhj1990y@gmail.com (H.J.L.); gowdavenu27@gmail.com (V.V.G.S.); ksm4234@naver.com (S.M.K.); sangdis2@naver.com (S.E.H.); 2 Gyeongnam Department of Environment Toxicology and Chemistry, Biological Resources Research Group, Korea Institute of Toxicology, 17 Jegok-gil, Jinju 52834, Korea; sjlee@kitox.re.kr (S.J.L.); jdher@kitox.re.kr (J.D.H.); 3 Department of Internal Medicine, Gyeongsang National University Cancer Center, School of Medicine, Gyeongsang National University, 15 Jinju-daero, Jinju 52727, Korea; suchibiodash@gmail.com (S.R.); lwshmo@gnu.ac.kr (W.S.L.); 4 Department of Nursing Science, International University of Korea, 965 Dongbu-ro, Jinju 52833, Korea; iuknurse@nate.com

**Keywords:** pectolinarigenin, gastric cancer, apoptosis, autophagy, PI3K/AKT/mTOR

## Abstract

Pectolinarigenin (PEC), a natural flavonoid present in *Cirsium chanroenicum* and in some species of *Citrus* fruits, has various pharmacological benefits such as anti-inflammatory and anti-cancer activities. In the present study, we investigated the anti-cancer mechanism of PEC induced cell death caused by autophagy and apoptosis in AGS and MKN28 human gastric cancer cells. The PEC treatment significantly inhibited the AGS and MKN28 cell growth in a dose-dependent manner. Further, PEC significantly elevated sub-G1 phase in AGS cells and G2/M phase cell cycle arrest in both AGS and MKN28 cells. Apoptosis was confirmed by Annexin V and Hoechst 33342 fluorescent staining. Moreover, Immunoblotting results revealed that PEC treatment down-regulated the inhibitor of apoptosis protein (IAP) family protein XIAP that leads to the activation of caspase-3 thereby cleavage of PARP (poly-ADP-ribose polymerase) in both AGS and MKN28 cells in a dose-dependent manner. The autophagy-inducing effect was indicated by the increased formation of acidic vesicular organelles (AVOs) and increased protein levels of LC3-II conversion in both AGS and MKN28 cells. PEC shows the down regulation of PI3K/AKT/mTOR pathway which is a major regulator of autophagic and apoptotic cell death in cancer cells that leads to the down-regulation of p-4EBP1, p-p70S6K, and p-eIF4E in PEC treated cells when compared with the untreated cells. In conclusion, PEC treatment might have anti-cancer effect by down-regulation of PI3K/AKT/mTOR pathway leading to G2/M phase cell cycle arrest, autophagic and apoptotic cell death in human gastric cancer cells. Further studies of PEC treatment can support to develop as a potential alternative therapeutic agent for human gastric carcinoma.

## 1. Introduction

Gastric cancer remains a predominant public health confrontation and a ferocious cause of death worldwide. Cancer-associated death has an extensive socio-economic burden globally, Republic of Korea being the first for gastric cancer-related deaths [1,2]. The most imperative etiological factors implicated in gastric carcinogenesis are *Helicobacter pylori* infection and diet [3]. The modern treatments such as chemotherapy and radiotherapy have their own limitations including drug resistance in cancers against anti-cancer drugs and adverse effects due to radiotherapy. Hence, there is an urgent need to establish an effective method to treat the cancer which is uncontrolled cell growth due to deregulation in the natural cell death mechanisms which eliminate mutated cells to develop as cancer cell and cancer progression without causing much destruction to normal cells.

Flourishing evidence indicates that autophagy affects distinct biological activities, such as cell survival, inflammatory responses, and apoptosis as well as implicated diseases, such as cancer, neurological disorders, and myocardial disease [4,5]. Autophagy represents a conserved process whereby nonessential intracellular components are transported to the lysosomes for degradation in response to a variety of stress stimuli, such as nutrient or growth factor deprivation, reactive oxygen species, damaged organelles, deoxyribonucleic acid (DNA) damage, hypoxia, protein aggregates, and intracellular microorganisms [5,6]. The role of autophagy in cancer is also paradoxical as it has dual roles in cell survival and death. Chemotherapy-induced autophagy stimulates a pro-survival response in cancer cells to develop drug resistance. Autophagy can inhibit apoptotic cell death by promoting cell survival; in contrast, autophagy and apoptosis can cooperate as partners to induce cell death [7,8]. Apoptosis is an evolutionary conserved and highly regulated cell death program that involves the suicide of cells in response to a number of stimuli, such as growth factor deprivation, antitumor drugs, and ionizing radiation, with the aim of preventing damage, stress, or the accumulation of non-functional cells in the tissue. Reduced caspase activation and elevated protein expression of inhibitor of apoptosis proteins (IAPs) lead to dysregulated apoptosis in cancer cells [9,10]. Overexpression of X-linked Inhibitor of Apoptosis (XIAP) has been shown to be associated with activated AKT in many cancers including gastric cancer. Up-regulation of AKT is involved in the conservation of XIAP degradation by chemotherapeutic agents in malignant cells [11,12,13]. mTOR, a key negative regulator of autophagy, is a serine/threonine protein kinase that modulates cell growth, cell proliferation, and protein synthesis. Down-regulation of AKT/PI3K leads to inactivated mTOR and induce autophagy in cancer cells [8,14,15]. Many studies have confirmed the PI3K/AKT/mTOR signaling pathway disorders in tumors, and particularly in the biological regulation of gastric, liver, breast, colorectal and prostate cancer cells. The pathway playing a role as proto-oncogene, which has become a hotspot of molecular biomarker-based and targeted therapy of tumors [16,17]. In cancer cells, PI3K/AKT activity is increased which activates mTOR complex via phosphorylation and decreases the feedback activation of p70S6k1/mTOR complex. These changes lead to increased and uncontrolled mitochondrial processes, ribosome biogenesis and angiogenesis for increased protein synthesis, cell proliferation, cell growth, and autophagy [18,19,20]. Regulating PI3K/AKT/mTOR pathway in cancer cells will be a key aspect to make cancer cell viable for cell death elimination using chemotherapeutic drugs which are nontoxic to normal cells.

Phytochemicals derived from plant sources have been regarded as an invaluable source of potential therapeutic agents, notably to target the multiple cellular signaling pathways such as apoptosis [21,22]. Flavonoids, a group of polyphenolic compounds derived from 2-phenylchromane, are found in ample quantities in vegetables, fruits, seed, peel, and tubers. Flavonoids have been reported to regulate cell death mechanisms by restoring the dysfunctional gene or activity that can promote massive cell death which was prevented from anticancer drugs [21,23]. Studies have reported that flavonoid induces autophagy and apoptotic cell death by altering key regulators such as PI3K/AKT/mTOR. Pectolinarigenin (PEC), a flavonoid compound, which is abundantly present in and can be isolated from *Cirsium chanroenicum* and some species of *Citrus* plant, has been shown to possess numerous pharmacological activities such as anti-inflammation, anti-cancer, and anti-allergy [24,25]. Research also reported PEC repressed cancer growth in vitro, including lung cancer, hepatocellular carcinoma, melanoma and colorectal adenocarcinoma [26,27]. However, the anti-cancer mechanism of PEC in gastric cancer is not well understood yet. Based on the above evidence, an investigation was undertaken to understand the mechanism of anti-cancer effects of PEC on human gastric cancer with AGS and MKN28 cells, focusing on cell death pathways.

## 2. Material and Methods

### 2.1. Chemicals and Antibodies

RPMI-1640 medium, fetal bovine serum (FBS) and antibiotics (Penicillin/Streptomycin) were purchased from Gibco (BRL Life Technologies, Grand Island, NY, USA). 3-(4,5-Dimethylthiazol-2-yl)-2,5-diphenyltetrazolium bromide (MTT) was obtained from Duchefa Biochemie (Haarlem, The Netherlands). Pectolinarigenin was purchased from AdooQ BioSience LLC (Irvine, KY, USA). Antibodies to caspase-3, -7, and -8; cleaved-caspase-3 and -7; poly ADP ribose polymerase (PARP); cleaved-PARP; p-AKT; p-4EBP1; p-p70S6K; p-eIF4E; mTOR; p-mTOR; LC3B; and Beclin-1 were purchased from Cell Signaling Technology (Danvers, MA, USA). XIAP, and AKT antibodies were obtained from Santa Cruz Biotechnology (Santa Cruz, CA, USA). β-actin was obtained from Millipore (Billerica, MA, USA). Annexin V fluorescein isothiocyanate (FITC) apoptosis detection kit was purchased from BD Pharmingen (San Diego, CA, USA). Propidium iodide (PI) was procured from Sigma-Aldrich (St. Louis, MO, USA). Hoechst 33342 trihydrochlorid stain was purchased from Invitrogen (Eugene, OR, USA). Materials and chemicals used for electrophoresis were obtained from Bio-Rad (Hercules, CA, USA).

### 2.2. Cell Culture and Cell Viability Assay

AGS (Well differentiated) and MKN28 (moderately differentiated) human gastric cancer cells were obtained from the Korea Cell Line Bank (Seoul, Korea) and were cultured in RPMI-1640 medium supplemented with 10% (*v*/*v*) heat-inactivated fetal bovine serum and 1% penicillin/streptomycin in a humidified atmosphere with 5% CO_2_ at 37 °C. To evaluate the effect of PEC on AGS and MKN28 cell growth, the cells were seeded at 1 × 10^6^ cells/mL in a 12-well plate and, were treated with PEC at various concentration (25, 50, 75, 100, 125 and 150 μM) and incubated for 24 h incubation at 37 °C. After incubation, 100 μL of MTT (0.5 mg/mL) were subsequently added to each well and incubated at 37 °C for 3 h. The culture medium was then removed and the purple formazan crystals produced by the mitochondrial dehydrogenase enzymes were dissolved in 500 μL of dimethyl sulfoxide (DMSO) and measured at 540 nm with an enzyme-linked immunosorbent assay (ELISA) plate reader (BioTek, Winooski, VT, USA).

### 2.3. Cell Cycle Distribution and Measurement of Cell Apoptosis

The AGS and MKN28 cells were incubated with or without PEC at 50 and 100 μM concentrations for 24 h at 37 °C. After incubation, the cells were washed with ice-cold PBS, trypsinized, collected in a 15 mL conical tube, and pelleted by centrifugation (1000× *g*) for 5 min. The pellets were washed twice with ice-cold PBS and cells were detached from the plates by trypsinization and fixed in 70% cold ethanol for 1 h at −20 °C. Prior to flow cytometric analysis, the cell solutions were centrifuged and the cells were washed in PBS, re-suspended in 400 μL of PBS containing 50 μg/mL Propidium iodide (PI) and 50 μg/mL RNase A and incubated in the dark for 15 min at room temperature. The DNA content per cell was analyzed using flow cytometry with Cytomics FC 500 (Beckman Coulter, Brea, CA, USA). In each sample, 10,000 cells were sorted. The data were analyzed using CXP Software (Beckman Coulter, Inc., Fullerton, CA, USA).

### 2.4. Annexin V Propidium Iodide Apoptosis Detection

Apoptotic cells were detected by using a FITC Annexin V apoptosis detection kit 1 according to the manufacturer’s protocol (BD Pharmingen, San Diego, CA, USA). Briefly, Cells were plated on six-well plates at 1 × 10^5^ cells/mL after treatment with various concentrations of PEC (0, 50 and 100 μM), for 24 h. The cells were collected and washed with PBS re-suspended in binding buffer. The cells were stained with Annexin V FITC and PI for 15 min at room temperature in the dark, prior to the addition of binding buffer. Flow cytometry analyses were performed with Cytomics FC 500 (Beckman Coulter, Brea, CA, USA). In total, 10,000 events per sample were sorted. The data were analyzed using CXP Software (Beckman Coulter, Inc., Fullerton, CA, USA).

### 2.5. Morphological Change and Hoechst 33342 Fluorescent Staining

AGS and MKN28 cells were seeded at density of 2 × 10^5^ per well and treated with indicated concentrations of PEC for 24 h at 37 °C. The cells were then washed with cold PBS and fixed with 37% Formaldehyde (1:4 dilutions with 95% Ethanol) for 10 min at room temperature. Subsequently, the fixed cells were washed with PBS and stained with Hoechst 33342 (2 μg/mL in PBS). The nuclear morphology of the cells was examined by fluorescence microscopy (EVOS^®^, Life Technologies).

### 2.6. Analysis of Acidic Vesicular Organelles (AVOs) with Acridine Orange Staining

After treatment with various concentrations of PEC (0, 50 and 100 μM) for 24 h, the cells were collected and washed with PBS and stained with acridine orange dye. Green (510 ± 530 nm) and red (650 nm) fluorescence emission from cells illuminated with blue (488 nm) excitation light were measured by flow cytometry analyses with BD Verse™ (BD Bioscience, Franklin Lakes, NJ, USA).

### 2.7. Western Blotting Analysis

For Western blotting analysis, both AGS and MKN28 cells were seeded in six-well plates at 2 × 10^5^ per well and treated with indicated concentrations of PEC (0, 50 and 100 μM) for 24 h at 37 °C. Cells were lysed in ice-cold RIPA buffer (1% (*w*/*v*) NP40, 1% (*w*/*v*) sodium deoxycholate, 0.1% (*w*/*v*) SDS, 0.15 M NaCl, 0.01 M sodium phosphate buffer, pH 7.2, 2 mM EDTA, and 50 mM phosphatase inhibitor cocktail). Protein lysates were centrifuged at 12,000 rpm for 30 min at 4 °C to remove insoluble material; protein concentrations were determined using a Pierce™ BCA assay (Thermo Fisher Scientific, Rockford, IL 61101, USA). Protein lysates resolved by electrophoresis on 10% or 12% SDS polyacrylamide gels; after gel electrophoresis, the proteins were electro transferred to a nitrocellulose membrane. Membranes were blocked with 5% non-fat skim milk in Tris-buffered saline containing 1% Tween 20 (TBS-T, pH 7.4) at room temperature for 1 h, and incubated overnight at 4 °C with a 1:1000 dilution of respected primary antibody. The membranes were washed five times with TBS-T for 10 min at room temperature, incubated with a 1:2000 dilution of HRP conjugated secondary antibody for 3 h at room temperature. Blots were developed under an ECL detection system (Bio-Rad Laboratory, Hercules, CA, USA). Protein quantification was performed using ImageJ (http://rsb.info.nih.gov). The densitometry readings of the bands were normalized according to β-actin expression.

### 2.8. RNA Purification and Quantification of mRNAs by Real-Time PCR

Total RNA was extracted from the gastric cancer cell lines using Trizol reagent (GeneALL, Biotechnology, Seoul, Korea) according to the manufacturer’s instructions after being treated with indicated concentrations of PEC for indicated times at 37 °C. The RNAs were quantified using a Nano Drop spectrophotometer. RNA was reverse-transcribed at 42 °C for 30 min using cDNA synthesis kits (Bio-Rad Laboratory, Hercules, CA, USA). The cDNA was subsequently amplified by PCR using the AccuPower^®^ 2X GreenStar™ qPCR MasterMix according to the manufacturer’s recommendation in a CFX96 Real-Time PCR system (Bio-Rad Laboratory, Hercules CA, USA). Relative fold levels were determined using β-actin genes as normalizer control. The primers (sequences: for p53, F: 5′-TGTCCGTCAGAACCCATGC-3′ and R: 5′-AAAGTCGAAGTTCCATCGCTC-3′; for p21, F: 5′-CAGCACATGACGGAGGTTGT-3′ and R: 5′-TCATCCAAATACTCCACACGC-3′; for β-actin, F: 5′-TACTGCCCTGGCTCCTCGCA-3′ and R: 5′-TGG ACAGTGAGGCCAGGATAG-3′) were purchased from Bioneer (Seoul, Korea).

### 2.9. Statistical Analysis

Differences between the groups were examined for statistical significance with Student’s *t*-test using SPSS Version 10.0 for Windows (SPSS, Chicago, IL, USA) and one-way ANOVA test were employed for data analysis. The results are expressed as the mean ± standard deviation (SD) of at least three independent experiments. A value of * *p* < 0.05 was considered significant.

## 3. Results

### 3.1. Pectolinarigenin (PEC) Inhibits Gastric Cancer Cell Growth in a Dose-Dependent Manner

**Figure 1:**
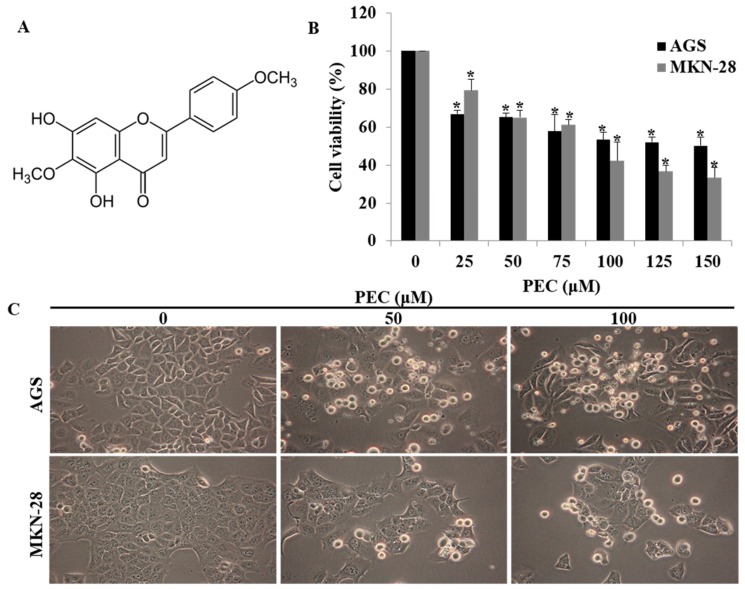
Effect of PEC on viability of human gastric cancer cells. (**A**) Structure of Pectolinarigenin (PEC). (**B**) AGS and MKN28 human gastric cancer cells were incubated with selected concentration of PEC (0–150 μM) for 24 h. Results indicated that PEC significantly inhibited human gastric cancer cell population growth in a dose-dependent manner compared to non-treated (control group) cells. Results are expressed as the mean ± standard deviation (SD) of at least three independent experiments. Statistical differences were analyzed with Student’s *t*-test (* *p* < 0.05 vs. control). (**C**) Morphology of AGS and MKN28 cells was examined under light microscopy (×400) after treated with PEC.

The chemical structure of pectolinarigenin (PEC) is shown in Figure 1A. The effect of PEC on cell viability was examined in gastric cancer cells AGS and MKN28 with MTT assay. As shown in (Figure 1B), PEC exhibited growth inhibitory effects on the cell lines with IC_50_ values in the sub-micromolar range after 24 h of treatment. The IC_50_ concentration in AGS cells and MKN28 cells were 124.79 μM and 96.88 μM, respectively. Therefore, we used 50 and 100 μM concentration of PEC in subsequent experiments. From morphology observations, 50 μM and 100 μM treatment of PEC for 24 h triggered massive cell rounding, shrinkage, and detachment from the culture plates (Figure 1C). Further, we investigated the underlying mechanism of PEC induced growth inhibition.

### 3.2. PEC Induced G2/M Phase Cell Cycle Arrest in AGS and MKN28 Cells and Involved Modulation of the Expression of Cell Cycle Regulators

**Figure 2:**
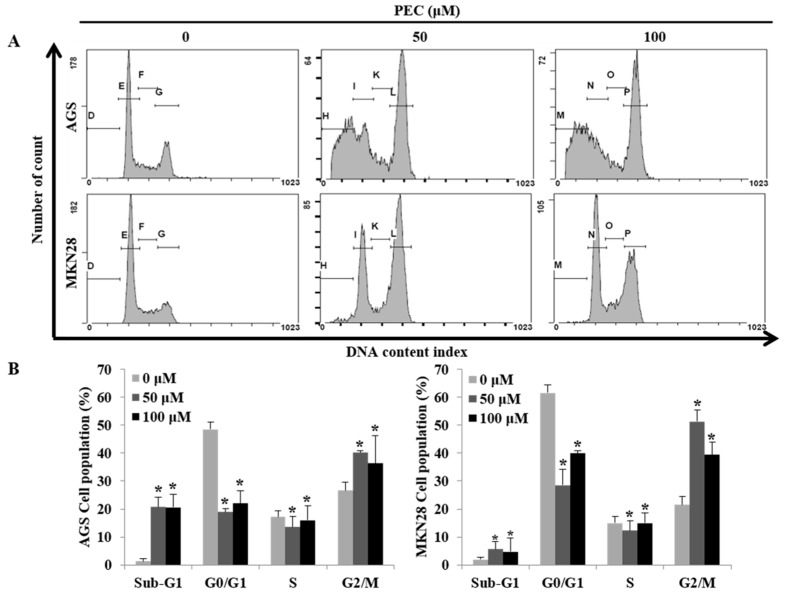

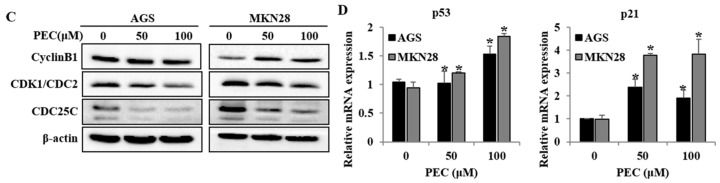
Flow cytometric analysis of cell cycle progression after PEC treatment and its regulatory mechanism. AGS and MKN28 cells were treated with indicated concentrations of PEC for 24 h. (**A**,**B**) Cell cycle distribution was determined by flow cytometry. The data were analyzed using CXP Software. (**C**) Effect of PEC on cell cycle-related proteins (cyclin B1, CDK1, and CDC25C) expression level in AGS and MKN28 cells. Cells were treated with PEC (0, 50 and 100 μM) for 24 h. Cell lysates were subjected to sodium dodecyl sulfate polyacrylamide gel electrophoresis (SDS-PAGE) and analyzed by Western blotting. Representative blots are shown. (**D**) qRT-PCR results show a significant difference in p53 and p21 mRNA levels in both AGS and MKN28 cells. The Bar indicates RNA expression normalized to β-actin. Results are expressed as the mean ± standard deviation (SD) of at least three independent experiments. Statistical differences were analyzed with Student’s *t*-test (* *p* < 0.05 vs. control).

Since PEC inhibited gastric cancer cell growth, we further examined whether PEC causes cell death by cell cycle arrest and/or apoptosis. Both AGS and MKN28 cells were treated with two different concentrations of PEC (50 and 100 μM) for 24 h and then the DNA content in cells was quantitated by flow cytometry of cells stained with Propidium iodide (PI). As shown in Figure 2A,B, there was a significant amount of cell accumulation at G2/M phase in both AGS and MKN28 cells treated with 50 and 100 μM and a slight increase in the sub-G1 phase of the cell population in MKN28, whereas significant amount of sub-G1 phase cells in AGS cells indicating apoptotic cell death population in AGS cells. To understand the possible molecular events associated with PEC-induced growth arrest in human gastric cancer cells, various cell cycle regulatory proteins were examined by Western blot analysis. Western blot results revealed that the expression of CDK1 and CDC25C protein were decreased in a dose-dependent manner, with significant inhibition occurring at 50 and 100 μM concentrations, as shown in Figure 2C. The qRT-PCR results revealed p21 and p53 expressions were significantly increased in mRNA levels in both cells (Figure 2D). There were no significant changes in the protein levels of p53 and p21 in both AGS and MKN28 cells treated with PEC (Western blot data not shown). Taken together, PEC caused growth arrest in the G2/M phase of cell cycle in both AGS and MKN28 cells by up-regulating p21 and p53 at mRNA levels and down-regulating CDK1 and CDC25C at protein level.

### 3.3. PEC Induces Apoptosis in AGS and MKN28 Cells

**Figure 3:**
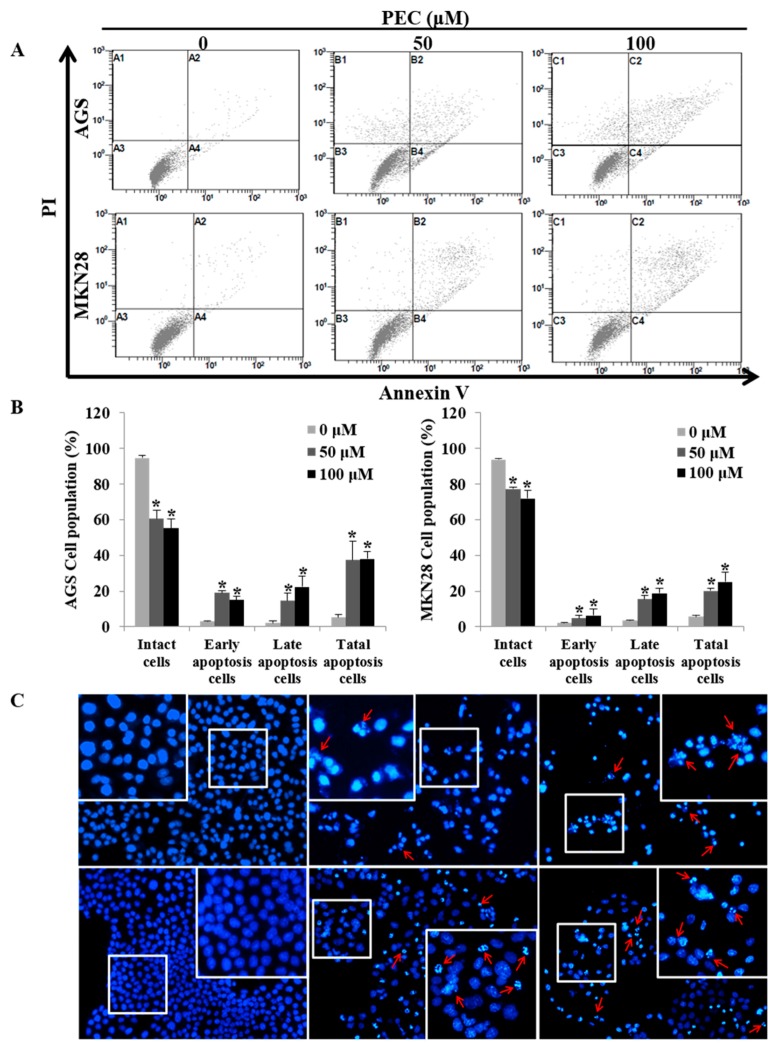
PEC induces dose-dependent apoptosis in AGS and MKN28 cells. (**A**,**B**) Cells were treated with indicated concentrations of PEC for 24 h. Apoptotic cells were stained with Annexin V/PI kit and determined by flow cytometry. The cells were analyzed using CXP Software. Results are expressed as the mean ± standard deviation (SD) of at least three independent experiments. Statistical differences were analyzed with Student’s *t*-test (* *p* < 0.05 vs. control). (**C**) Both AGS and MKN28 cells were stained with Hoechst 33342 and examined under fluorescence microscopy (×400). (Red arrows showing bright blue regions indicate fragmented or condensed nuclei).

**Figure 4:**
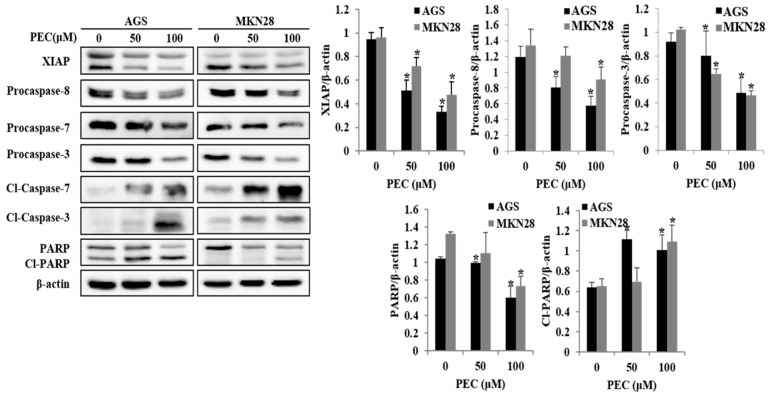
Effects of PEC concentration on the expression levels of XIAP, caspase-3/7 activation, and cleavage of PARP in AGS and MKN28 cells. AGS and MKN28 cells were treated with indicated concentrations of PEC for 24 h. The cell lysates were subjected to SDS-PAGE and analyzed by immune-blotting. Densitometry analyses of XIAP, Procaspase-8, Procaspase-7, Procaspase-3, cleaved caspase-7, cleaved caspase-3 and cleaved PARP proteins expressions are expressed as the mean ± standard deviation (SD) of at least three independent experiments. Statistical differences were analyzed with Student’s *t*-test (* *p* < 0.05 vs. control).

Since apoptotic cells with hypodiploid DNA content were detected in the sub-G1 phase of cell cycle, translocation of membrane phosphatidylserine (PS) was evaluated to confirm whether PEC induces apoptosis. Annexin V-FITC/PI assay using flow cytometry in both AGS and MKN28 cells were treated with indicated concentration of PEC for 24 h. Cytometric results revealed in (Figure 3A,B) shows that PEC induced apoptotic cell population in both AGS and MKN28 cells in a dose-dependent manner. After 24 h exposure to PEC, the increasing dose resulted in an increased proportion of total apoptotic cell by more than six folds in 100 μM PEC treated cells compared to control group of cells; the early apoptotic population is major in AGS cells. Whereas MKN28 cells after 24 h treatment, increased dosage resulted in an increased proportion of total apoptotic cell by more than five folds in 100 μM PEC treated cells compared to control group of cells; the late apoptotic population is being major in MKN28 cells. To confirm PEC induced cell death is apoptotic cell death, nuclear staining was conducted on both the cells with Hoechst 33342 Fluorescent Staining after 24 h of PEC treatment. As shown in Figure 3C, bright blue regions indicate fragmented or condensed nuclei in the treated group of cells, thereby confirming that PEC is causing the apoptotic cell death. Furthermore, as shown in Figure 4, treatment of AGS and MKN28 cells with various concentrations of PEC (0, 50, and 100 μM) for 24 h resulted in a significant decrease of procaspases-8, -7 and -3, while activating cleaved caspases-3 and -7 significantly increased, which further leads to the cleavage of PARP. The down-regulation of IAP family protein XIAP leads to significant activation of caspase-3 in both AGS and MKN28 cells in a dose-dependent manner when compared between untreated and PEC treated group of cells. Thus, these results demonstrated the ability of PEC to induce cell death in AGS and MKN28 cells through apoptosis.

### 3.4. PEC Induces Autophagy in AGS and MKN28 Cells

**Figure 5:**
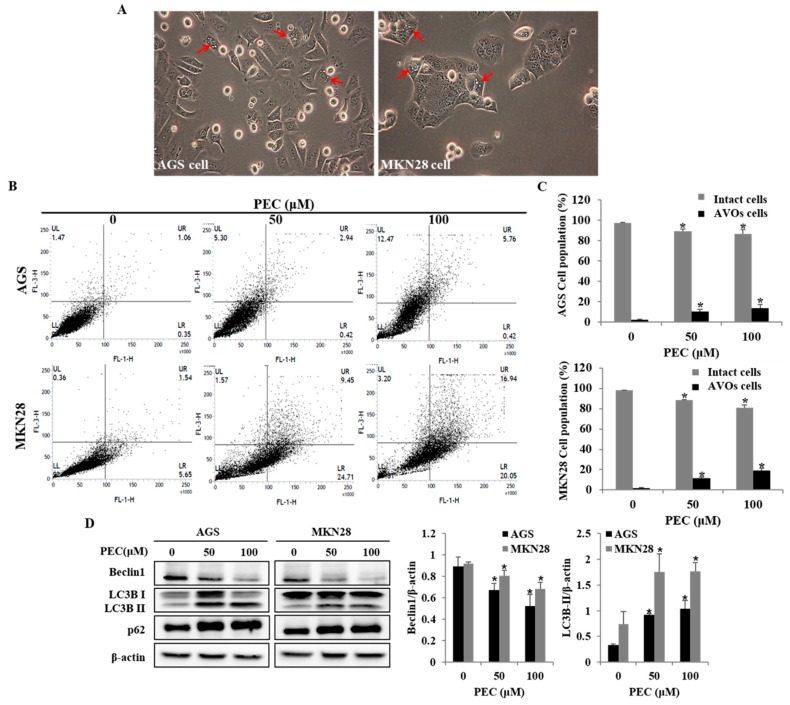
PEC induces autophagy cell death in AGS and MKN28 cells. (**A**) Morphology of both AGS and MKN28 cells was examined under light microscopy (×400) after treated with PEC (100 μM). (The red arrows indicate the autophagic vacuoles.) (**B**) The cells were treated with PEC at 0, 50 and 100 μM concentrations for 24 h. After incubation, the cells were stained with 5 μg/mL acridine orange for 15 min and collected in phenol red-free growth medium. Green (510 ± 530 nm) and red (650 nm) fluorescence emission illuminated with blue (488 nm) excitation light were measured with a flow cytometer. PEC induced dose-dependent AVO formation in AGS and MKN28 cells. (UR and UL: AVOs cells; LL and LR: Intact cells). (**C**) AGS and MKN28 were treated with indicated concentrations of PEC or 24 h. The cell lysates were subjected to SDS-PAGE and analyzed by immune-blotting. Densitometry analyses of Beclin1, LC3, and p62 protein expressions are presented. Results are expressed as the mean ± standard deviation (SD) of at least three independent experiments. Statistical differences were analyzed with Student’s *t*-test (* *p* < 0.05 vs. control).

To investigate whether PEC treatment induced autophagy in AGS and MKN28 cells, the autophagic cell death was examined. Autophagy markers microtubule-associated protein1 light chain3 (LC3), p62 and Beclin-1 were analyzed with Western blot. During autophagy, a cytosolic form of LC3 (LC3-I) is conjugated to phosphatidylethanolamine to form membrane-bound LC3 (LC3-II). In this study, two variants of LC3 were detected, wherein the ratio of LC3-II/LC3-I was shown to increase in a dose-dependent manner; meanwhile, decreased expression was observed for Beclin-1 in both AGS and MKN28 cells (Figure 5D). PARP-1 activation is essential in the autophagy process during the response to the chemotherapeutic agent. In the current results, it was observed (Figure 4) that PEC induced cleavages of PARP in a dose-dependent manner. In addition, increased formation of AVOs is one of the characteristics of autophagy. Hence, the formation of AVOs in PEC-treated AGS and MKN28 cells were analyzed by flow cytometry with Acridine Orange (AO) dye, which supported dose-dependent PEC-induced accumulation of AVOs (Figure 5B,C). Taken together, the results suggest that PEC induced Beclin-1-independent autophagy by promoting the conversion of LC3-I to LC3-II followed by PARP activation and cleavage in AGS and MKN28 cells.

### 3.5. Effects of PEC on PI3K/AKT/mTOR Signaling Pathway for the Induction of Cell Cycle Arrest, Autophagy, and Apoptosis in AGS and MKN28 Cells

**Figure 6:**
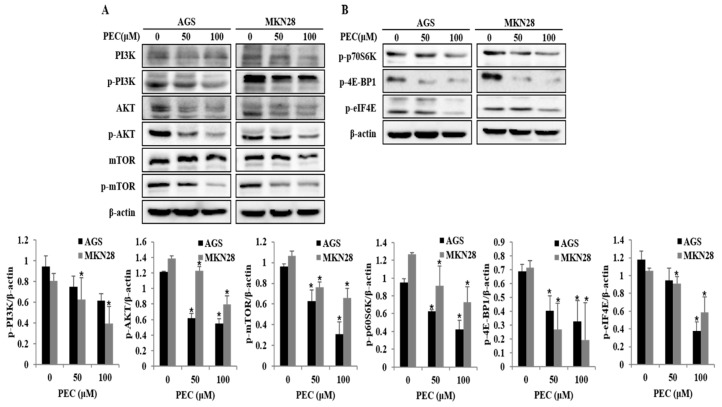
Effect of PEC on inhibition of PI3K/AKT/mTOR pathway. (**A**,**B**) AGS and MKN28 were treated with indicated concentrations of PEC or 24 h. The cell lysates were subjected to SDS-PAGE and analyzed by immune-blotting. Densitometry analyses of p-PI3K, p-AKT, p-mTOR, p-p70S6K, p-4E-BP1 and p-eIF4E protein expressions are presented as the mean ± standard deviation (SD) of at least three independent experiments. Statistical differences were analyzed with Student’s *t*-test (* *p* < 0.05 vs. control).

PI3K/AKT/mTOR signaling is one of the comprehensive pathways activated in most cancers, including gastric cancer. This pathway plays a diversity of physiological roles, including regulation of cell growth, cell cycle and cell survival. Recent studies have demonstrated that inhibition of PI3K/AKT/mTOR pathway is associated with triggering autophagy and apoptotic cell death in cancer cells. As shown in Figure 6A,B, treatment with PEC reduced the expression PI3K after 24 h of treatment and, at the same time, a decrease in the phosphorylation (at Ser473) of AKT protein is observed. We further investigated the effect of PEC treatment on mTOR activity. Exposure of AGS and MKN28 cells to PEC resulted in diminished levels of phosphorylated (activated) form of mTOR (Ser2448). PEC treatment also induced an acute decrease in the phosphorylation of the mTOR targets p70 ribosomal protein S6 kinase (p-p70S6K), 4E-BP1 and p-eIF4E, revealing a potent inhibitory effect of PEC on PI3K/AKT/mTOR signaling leading to cell cycle arrest, autophagy and apoptosis in human gastric cancer cells treated with PEC.

## 4. Discussion

Increasing evidence indicates that herbal extracts and their compounds, especially flavonoids, can inhibit cell growth and promote cancer cell death through different mechanisms including cell cycle arrest, autophagy and apoptosis [28,29]. In our recently published study, we showed that flavonoids extract from Korean *Citrus platymamma hort. ex Tanaka* (FCP) containing flavonoids such as Naringin, Hesperidin, and PEC suppressed the cell viability and induced caspase-dependent cell death in human gastric cancer cells [30]. Importantly, *Citrus platymamma hort*. has shown to be safe for consuming by both humans and animals. In the present study, we examined the potential anticancer activity of PEC on AGS, a well differentiated adenocarcinoma gastric cancer cells from a 54-year-old Caucasian female stomach, and MKN28, a moderately differentiated tubular adenocarcinoma gastric cancer cells derived from a 70-year-old Mongolian female. Our findings exhibit that PEC inhibits the viability of human gastric cancer cells through inactivation of proteasome-dependent degradation of Akt/PI3K/mTOR pathway. Indeed, we found that PEC treatment first stimulates the intracellular protein ubiquitination and proteasome degradation of proteins including caspase-3/-7 and AKT in a concentration-dependent manner. Furthermore, we observed non-canonical Beclin-1-independent autophagy and subsequent caspase-dependent apoptosis that ultimately leads to the death of gastric cancer cells. Apoptosis is accompanied with a reduction of the G1 phase cells and an increase in the G2/M phase cells, signifying a cell cycle arrest in G2/M phase. Moreover, similar results were observed with previous studies showing that an increase in the number of G2/M phase cells is clearly related to apoptosis [31,32]. In the current study, we found that PEC treatment decreased the percentage of G1 phase and increased sub-G1 and G2/M phase cells. These observations could be the results of apoptotic response in AGS and MKN28 cells treated with PEC. Besides, in the Hoechst 33342 staining, apoptotic particles were observed confirming PEC induces apoptosis in human gastric cancer cells. As caspases play a pivotal role in the apoptotic cell death, it has been reported that initiator caspase-8 and -9 activate caspase-3 and -7 directly or indirectly and can be blocked by XIAP. We were not surprised to find that PEC regulated caspase family by decrease of XIAP, procaspase-8, and -3, and an increase of cleaved caspase-3, cleaved caspase-7 edge to cleavage of PARP indicating apoptotic signal pathway.

The PI3K/AKT/mTOR pathway is one of the comprehensive signaling pathways that regulate cell proliferation, growth, metabolism, and cell survival and is one of the most periodically deregulated pathways in cancer. A well-defined upstream regulator of mTORC1 is the phosphoinositide 3-kinase-AKT-mammalian target of rapamycin (PI3K/AKT/mTOR) signaling pathway, although the activation of mTORC1 can also occur through an AKT-independent pathway. Activated AKT directly phosphorylates and consequently inhibits TSC1/2 leading to activation of mTORC1. Activated mTOR coordinates the overall protein turnover and thus promoting cell growth and proliferation. In addition, mTOR pathway negatively regulates autophagy, and the inactivation of mTOR is considered a crucial step in autophagy activation. The PI3K/AKT/mTOR pathway is frequently hyperactivated in many cancers, including gastric cancer, and is important for aggressive tumor growth and cell survival. Recently, inhibition of this pathway through genetic/chemical inhibition of mTORC1 has emerged as a potential target for gastric cancer. Aberrant activation of the PI3K/AKT/mTOR signaling pathway through loss of the tumor suppressor PTEN protein or mutations in proteins in the PI3K/AKT pathway are extremely common in gastric cancer and other types of cancer [16]. In the present study, PEC significantly decreased the expression levels of p-PI3K, p-AKT (Ser473), which led to down-regulation of p-mTOR, showing the ability of PEC to inhibit the hyperactivated PI3K/AKT/mTOR in human gastric cancer cells. Protein eIF4E levels are rate limiting; phosphorylation of 4E-BPs during aberrant mTORC1 activation in cancer is a strong inducer of cap-dependent translation. Consequential activation of mTOR signaling and increased activity of eIF4E cap-dependent translation is thought to be a critical mediator for tumorigenesis [33]. eIF4E is an oncogene as it is overexpressed in many human cancers with poor prognosis and its overexpression results in the transformation of cells in vivo. Stimulation of protein synthesis occurs through mTOR increases in ribosome biogenesis, and direct phosphorylation of p70S6K (ribosomal protein S6 kinase), 4E-BPs (eIF4E-binding proteins) and eEF2K (eukaryotic elongation factor 2), resulting in increased rates of initiation and elongation, and increased capacity of mRNA translation. Exalted phosphorylation of 4E-BPs in cancer cells, also results in poor patient prognosis and the down-regulation of 4E-BP1 with the resulting activation of cap-dependent translation encourage cell proliferation and cell-cycle progression in cell culture [33,34,35]. In the present study, down-regulation of p-mTOR led to inhibition of p-p70S6K (Thr389) followed by p-4EBP1 (Thr37/46) in AGS and MKN28 cells treated with PEC. Hence, the results suggest that PEC can act has PI3K/AKT/mTOR pathway inhibitor to regulate the cell cycle arrest, autophagy and apoptosis cell death in PEC treated human gastric cancer cells.

**Figure 7:**
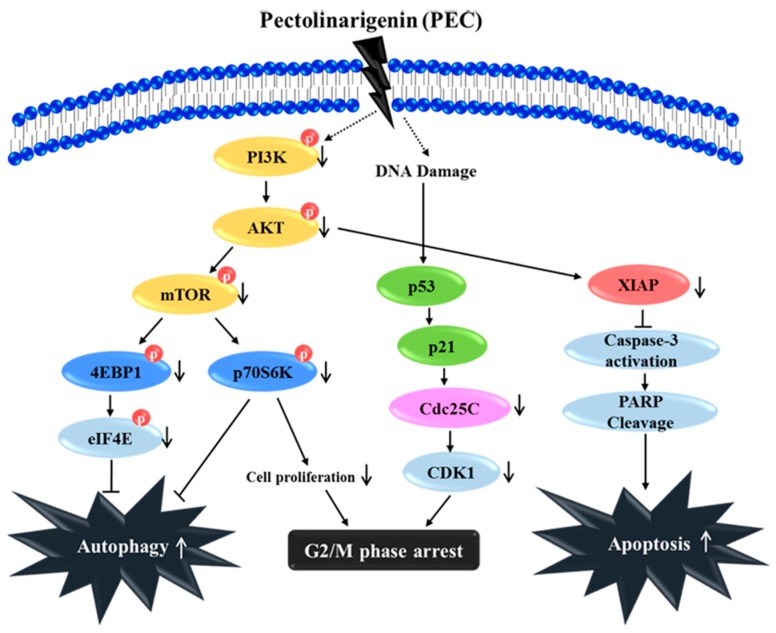
Schematic diagram characterizes the anti-cancer mechanism of the PEC treatment in both AGS and MKN28 cells. PEC induced G2/M phase arrest, autophagy, and apoptosis by down-regulation of PI3K/AKT/mTOR pathway, leading to the down-regulation of p-4E-BP1, p-p70S6K, and p-eIF4E. Altogether, PEC treatment has potential as a chemotherapeutic for the treatment of human gastric carcinoma.

In summary, our study has elucidated a novel anti-cancer mechanism of PEC in AGS and MKN28 human gastric cancer cells to induce G2/M arrest, apoptosis, and autophagy in vitro (Figure 7). Our results show that the PI3K/AKT/mTOR pathway plays a vital role in PEC induced cell death in human gastric cancer cell. PEC could be a new anticancer therapeutic agent enhancing the treatment responses for human gastric carcinoma with potential impact on the choice of therapy in the case of inhibitors of PI3K, AKT, and mTOR, which is cancer specific. Although PEC is known for its anticancer effects, to the best of our knowledge, this is the first study on the molecular mechanisms of PEC against human gastric cancer AGS and MKN28 cells. Further study will be undertaken to explore the combination therapy and comparison with current chemotherapeutic drugs of gastric cancer with PEC, which will increase the translation relevance for considering PEC as anti-cancer agent.
